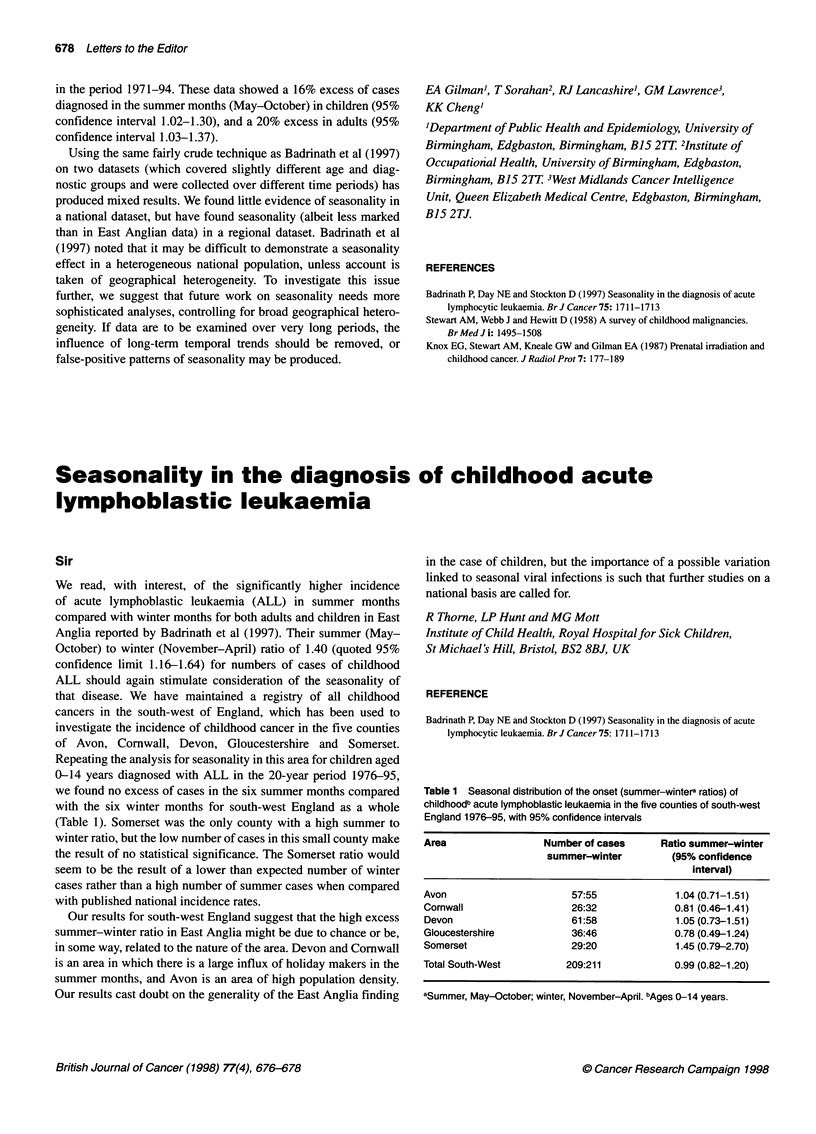# Seasonality in the diagnosis of childhood acute lymphoblastic leukaemia.

**DOI:** 10.1038/bjc.1998.110

**Published:** 1998-02

**Authors:** R. Thorne, L. P. Hunt, M. G. Mott


Badrinath P, Day NE and Stockton D (1997) Seasonality in the diagnosis of acute lymphocytic leukaemia. *Br J Cancer* **75**: 1711–1713

Stewart AM, Webb J and Hewitt D (1958) A survey of childhood malignancies. *Br Med J* **i**: 1495–1508

Knox EG, Stewart AM, Kneale GW and Gilman EA (1987) Prenatal irradiation and childhood cancer. *J Radiol Prot* **7**: 177–189

### Seasonality in the diagnosis of childhood acute lymphoblastic leukaemia

**Sir**

We read, with interest, of the significantly higher incidence of acute lymphoblastic leukaemia (ALL) in summer months compared with winter months for both adults and children in East Anglia reported by Badrinath et al (1997). Their summer (May–October) to winter (November–April) ratio of 1.40 (quoted 95% confidence limit 1.16–1.64) for numbers of cases of childhood ALL should again stimulate consideration of the seasonality of that disease. We have maintained a registry of all childhood cancers in the south-west of England, which has been used to investigate the incidence of childhood cancer in the five counties of Avon, Cornwall, Devon, Gloucestershire and Somerset. Repeating the analysis for seasonality in this area for children aged 0–14 years diagnosed with ALL in the 20-year period 1976–95, we found no excess of cases in the six summer months compared with the six winter months for south-west England as a whole (Table 1). Somerset was the only county with a high summer to winter ratio, but the low number of cases in this small county make the result of no statistical significance. The Somerset ratio would seem to be the result of a lower than expected number of winter cases rather than a high number of summer cases when compared with published national incidence rates.

Our results for south-west England suggest that the high excess summer–winter ratio in East Anglia might be due to chance or be, in some way, related to the nature of the area. Devon and Cornwall is an area in which there is a large influx of holiday makers in the summer months, and Avon is an area of high population density. Our results cast doubt on the generality of the East Anglia finding in the case of children, but the importance of a possible variation linked to seasonal viral infections is such that further studies on a national basis are called for.

*R Thorne, LP Hunt and MG Mott*
*Institute of Child Health, Royal Hospital for Sick Children, St Michael's Hill, Bristol, BS2 8BJ, UK*

**REFERENCE**

Badrinath P, Day NE and Stockton D (1997) Seasonality in the diagnosis of acute lymphocytic leukaemia. *Br J Cancer* **75**: 1711–1713

**Table 1** Seasonal distribution of the onset (summer–winter[a] ratios) of childhood[b] acute lymphoblastic leukaemia in the five counties of south-west England 1976–95, with 95% confidence intervals

| Area | Number of cases summer–winter | Ratio summer–winter (95% confidence interval) |
|---|---|---|
| Avon | 57:55 | 1.04 (0.71–1.51) |
| Cornwall | 26:32 | 0.81 (0.46–1.41) |
| Devon | 61:58 | 1.05 (0.73–1.51) |
| Gloucestershire | 36:46 | 0.78 (0.49–1.24) |
| Somerset | 29:20 | 1.45 (0.79–2.70) |
| Total South-West | 209:211 | 0.99 (0.82–1.20) |

[a]Summer, May–October; winter, November–April. [b]Ages 0–14 years.

*© Cancer Research Campaign 1998*